# Common bean resistance to *Xanthomonas* is associated with upregulation of the salicylic acid pathway and downregulation of photosynthesis

**DOI:** 10.1186/s12864-020-06972-6

**Published:** 2020-08-18

**Authors:** Justine Foucher, Mylène Ruh, Anne Préveaux, Sébastien Carrère, Sandra Pelletier, Martial Briand, Rémy-Félix Serre, Marie-Agnès Jacques, Nicolas W. G. Chen

**Affiliations:** 1 IRHS, INRAE, AGROCAMPUS OUEST, Université d’Angers, SFR4207 QUASAV, 42, rue Georges Morel, F-49071 Beaucouzé, France; 2 CNRS, UMR 2594, Laboratoire des Interactions Plantes-Microorganismes (LIPM), F-31326 Castanet-Tolosan, France; 3 INRAE, US 1426, GeT-PlaGe, Genotoul, Castanet-Tolosan, France

**Keywords:** Common bean, *Xanthomonas*, Common bacterial blight, RNA-Seq, Resistance

## Abstract

**Background:**

Common bacterial blight (CBB) caused by *Xanthomonas phaseoli* pv. *phaseoli* and *Xanthomonas citri* pv. *fuscans* is one of the major threats to common bean crops (*Phaseolus vulgaris* L.). Resistance to CBB is particularly complex as 26 quantitative resistance loci to CBB have been described so far. To date, transcriptomic studies after CBB infection have been very scarce and the molecular mechanisms underlying susceptibility or resistance are largely unknown.

**Results:**

We sequenced and annotated the genomes of two common bean genotypes being either resistant (BAT93) or susceptible (JaloEEP558) to CBB. Reciprocal BLASTp analysis led to a list of 20,787 homologs between these genotypes and the common bean reference genome (G19833), which provides a solid dataset for further comparative analyses. RNA-Seq after inoculation with *X. phaseoli* pv. *phaseoli* showed that the susceptible genotype initiated a more intense and diverse biological response than the resistant genotype. Resistance was linked to upregulation of the salicylic acid pathway and downregulation of photosynthesis and sugar metabolism, while susceptibility was linked to downregulation of resistance genes and upregulation of the ethylene pathway and of genes involved in cell wall modification.

**Conclusions:**

This study helps better understanding the mechanisms occurring during the early colonization phase of common bean by *Xanthomonas* and unveils new actors potentially important for resistance and susceptibility to CBB. We discuss the potential link between the pathways induced during bean colonization and genes induced by transcription activator-like effectors (TALEs), as illustrated in other *Xanthomonas* pathovars.

## Background

Plant immunity is governed by a two-tier system capable of monitoring the presence of pathogens [1, 2]. The first layer of the plant immune system consists of the recognition of evolutionarily conserved pathogen- or microbe-associated molecular patterns (PAMPs or MAMPs) by pattern-recognition receptors (PRRs) [3, 4]. PRRs belong typically to the receptor-like kinase (RLK) family, which encompasses membrane proteins with an extracellular domain composed of either leucine-rich repeats or lysin motifs [5]. Activation of PRRs leads to a complex response called PAMP-triggered immunity (PTI) involving intracellular signaling, transcriptional reprogramming, and biosynthesis of compounds that limit microbial colonization [6]. Pathogens employ an array of proteins called effectors, a large proportion of which are able to subvert PTI [7]. For example, bacterial pathogens use a type III secretion system to inject dozens of type III effectors into host cells [8–10]. The second layer of plant immunity or effector-triggered immunity (ETI) consists of the direct or indirect recognition of effectors by intracellular disease resistance (*R*) proteins [11]. Most of these proteins belong to a large family of nucleotide-binding leucine-rich repeat (NLR) receptors. After pathogen detection, plants usually trigger a burst of Ca^2+^ followed by an extracellular production of reactive oxygen species and activation of mitogen-activated protein kinases (MAPKs). Different molecular pathways can be activated, involving major hormones such as salicylic acid (SA), jasmonic acid (JA) and ethylene, which are key for regulating the immune response to pathogen [12–14]. These pathways lead to defense responses characterized by, but not limited to the production of pathogenesis-related (PR) proteins and cell wall reinforcement [15].

Common bean (*Phaseolus vulgaris* L.) is the main grain legume used for direct human consumption. This crop is of particular interest for human nutrition, as it is one of the main sources of protein in many countries from America and Africa, as well as a source of vitamins, fibers and minerals [16, 17]. Common bean originated from Mesoamerica, from where populations migrated to the Andean region 146,000 to 184,000 years ago, resulting in the creation of two major gene pools (Mesoamerican and Andean) [18]. Wild plants from both gene pools differ in morphology, Mesoamerican having thinner leaves and producing pods and seeds that are smaller, though more numerous than their Andean counterpart [19].

Common bacterial blight of bean (CBB) is one of the main threats to bean cultivation with yield losses of more than 40% under favourable conditions [20, 21] and up to 75% in the most severe cases [22, 23]. CBB is characterized by water-soaking spots on leaves, stems and pods, further evolving into necrotic lesions sometimes surrounded by a chlorotic halo in leaves. This disease is caused by *Xanthomonas phaseoli* pv. *phaseoli* and *Xanthomonas citri* pv. *fuscans* [24–27]. These bacteria are mainly transmitted by seeds, and occur in all regions where common bean is cultivated [22]. CBB is mainly controlled by prophylactic methods such as the use of pathogen-free seeds, two-year rotations with non-leguminous crops and burning of plant residues [28]. In quarantine areas, seed lots are routinely tested using a method involving isolation of bacterial strains and identification using specific PCR or pathogenicity tests [29, 30]. However, *X. citri* pv. *fuscans* and *X. phaseoli* pv. *phaseoli* were recently removed from the quarantine list of the European Union (EU delegated regulation 2019/2072), which was previously the main quarantine zone for CBB agents worldwide. In this context, it is important to develop and use resistant genotypes, which is the most economic and ecologically safe management strategy against CBB [31, 32].

Although variations of CBB resistance levels have been observed in several common bean accessions, no major *R* gene to CBB has been described so far [33–35]. On the other hand, several *R* gene to CBB have been described in *P. acutifolius* and *P. coccineus* [36, 37]. Introgression of CBB resistance from *P. acutifolius* in XAN lines led to two major quantitative resistance loci (QRLs) associated with markers SU91 and BC420 [38, 39], which explain a significant part of the phenotypic variation [40–42]. Most resistant cultivars bred for CBB resistance possess the SAP6 marker deriving from the Great Northern landrace cultivars, such as ‘Montana No.5’ and ‘GN #1 sel 27’ [43, 44]. So far, 26 QRLs to CBB have been mapped using nine different bi-parental populations. These QRLs are dispersed throughout the common bean genome and poorly co-localize with each other when comparing the different populations tested. Moreover, most CBB resistances vary according to plant maturity, plant organs (leaf, pod or seed), pathogenic strains and environment [34, 45, 46], which reflects the high complexity of CBB resistance.

Analyzing the transcriptomic response of common bean to CBB is a way to enhance our knowledge on the molecular mechanisms underlying CBB resistance, and can provide important information for developing genetic management of the disease. However, to our knowledge, transcriptomic studies during *X. phaseoli* pv. *phaseoli* infection are limited to a cDNA-AFLP analysis [47] and RT-qPCR assays focused on *R* genes [48, 49]. Previous work using *X. phaseoli* pv. *phaseoli* isolate W18 identified four QRLs to CBB in a cross between the Mesoamerican genotype BAT93 (resistant to CBB) and the Andean genotype JaloEEP558 (susceptible to CBB) [50]. These QRLs are located on chromosomes 2, 5, 7 and 9 and explain 75% of the phenotypic variation. Here, we studied the transcriptomic response of BAT93 and JaloEEP558 to *X. phaseoli* pv. *phaseoli* strain CFBP6546R 48 h after inoculation.

## Results

### Pathogenicity assays

**Fig. 1:**
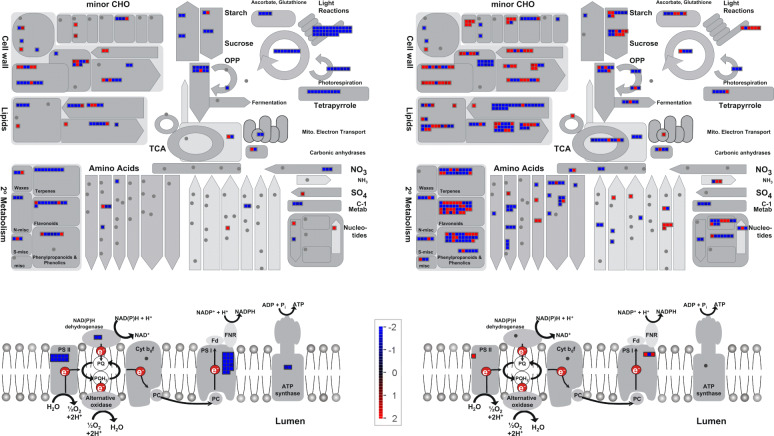
Pathogenicity of strain CFBP6546R on BAT93 and JaloEEP558. Bacterial population sizes over time on BAT93 (dotted line) and JaloEEP558 (full line) (**a**). Quantification of symptoms assessed by chlorophyll fluorescence imaging at 8 and 15 DPI (**b**). Total symptomatic areas corresponded to the sum of impacted, wilted and necrotic tissues defined by using Fv/Fm tresholds as previously compared to visual inspection [52]. Error bars represent the standard errors of the means for three biological replicates. Below the histogram are examples of the leaflets presenting symptoms representative of each condition, obtained by chlorophyll fluorescence imaging (top) or visible imaging (bottom). Letters indicate significantly different groups (Mann-Whitney test, *p*-value < 0.05). CFU: colony-forming units. gFM: grams of fresh materials. DPI: days post inoculation

Resistance and susceptible phenotypes of common bean genotypes BAT93 and JaloEEP558 were demonstrated with bacterial growth and symptom development after inoculation with *X. phaseoli* pv. *phaseoli* strain CFBP6546R. For both genotypes, bacterial population sizes decreased during the first day, then increased rapidly up to day 5 and stabilized over time, which is a typical dynamic for common bean colonization by *Xanthomonas* [51]. However, bacterial population sizes were significantly higher on JaloEEP558 than on BAT93 (*p*-value < 0.05) at 8 and 15 days post inoculation (Fig. 1A). Moreover, BAT93 presented almost no symptoms, while 39 to 50% of the leaf area were symptomatic on JaloEEP558 (Fig. 1B). Symptoms 15 days post inoculation (DPI) were characterized by the apparition of necrotic areas that were not detected at 8 DPI. These results show that JaloEEP558 is susceptible, while BAT93 is resistant to strain CFBP6546R, as previously described for *X. phaseoli* pv. *phaseoli* isolate W18 [50].

### Whole genome sequencing and annotation

The genomes of BAT93 and JaloEEP558 were sequenced and annotated to serve as basis for the mapping of corresponding RNA-Seq reads. For each genotype, Illumina sequencing produced around 3.2 × 10^8^ paired reads totalizing 6.4 × 10^4^ Mbp. The resulting assembly consisted of 36,622 scaffolds totalizing 453.0 Mbp for BAT93, and 31,483 scaffolds totalizing 449.3 Mbp for JaloEEP558, with a coverage of approximately 100X for both genotypes (Table 1). These assemblies represented around 84% of the *P. vulgaris* v2.1 reference genome (537.2 Mbp) from the Andean genotype G19833 [18] or 77% of the estimated genome size (~ 587 Mbp), and 82% of the BAT93 reference genome (549,6 Mbp) published by Vlasova et al. in 2016 [53]. Conversely, structural annotation predicted 33,275 and 32,914 protein coding genes for BAT93 and JaloEEP558, respectively, which are numbers higher than those found in the reference genomes of G19833 (27,433) or BAT93 (30,491). Reciprocal BLASTp (e-value ≤1 × 10^− 6^) using all predicted genes showed that our BAT93 and JaloEEP558 genome sequences shared more homologs (27,208) together than with both G19833 and BAT93 reference genomes (Table S1). Thus, to avoid possible biases due to different sequencing and annotation methods, we used our own version of the BAT93 genome for RNA-Seq mapping and comparative transcriptomic analysis between BAT93 and JaloEEP558. To ensure the quality of our data, we used a subset of homologs between BAT93 and JaloEEP558 that had the same best hit after BLASTp on the G19833 reference genome. After removal of paralogs, we obtained 20,787 unique genes that we used for all the analyses presented hereafter (Table S2).

**Table 1:** Summary of sequencing, assembly and annotation data

	BAT93	JaloEEP558
**Whole genome sequencing**		
Assembly length (bp)	452,993,439	449,275,055
Coverage	108x	114x
Number of scaffolds	36,622	31,483
N50 (size/number)	35,794/3086	44,310/2481
N90 (size/number)	4951/16,296	5936/13,165
% of Ns	0.92%	0.84%
**Annotation**		
Predicted genes	33,275	32,914
**RNA sequencing**		
Average raw reads	23,237,717	23,040,441
Average mapped reads on genes	20,197,275	20,731,141
% of mapped reads on genes	87%	90%

### Analysis of differentially-expressed genes

To explore the responses of BAT93 and JaloEEP558 to strain CFBP6546R, transcriptomes of inoculated leaves were produced. In other *Xanthomonas*-plant pathosystems such as tomato [54], rice [55] or sweet orange [56, 57], bacterial effectors impacted plant transcriptomes around 24 to 48 h after leaf infiltration. Here, we performed RNA-Seq analyses on inoculated leaves 48 h after infiltration. Illumina sequencing led to a total of 139.4 and 138.2 million raw reads for BAT93 and JaloEEP558 respectively, with an average of 23.2 million raw reads per sample for BAT93 and 23.0 million raw reads per sample for JaloEEP558 (Table 1). After stringent quality check, data cleaning and mapping, we obtained an average of 20.2 million mapped reads on genes per sample (87% of raw reads) for BAT93 and 20.7 million mapped reads on genes per sample (90% of raw reads) for JaloEEP558. Water-inoculated and bacteria***-***inoculated plants formed distinct groups after principal component analysis, confirming the similarity of biological replicates within each condition and the similarity of both genotypes after water treatment (Fig. S1). Similar trends were observed using a Pearson correlation matrix, indicating that the bacteria had a significant effect on the transcriptomes of both genotypes. After bacterial inoculation, a total of 5581 out of the 20,787 homologs were differentially expressed in at least one genotype compared to water inoculation (Table S3), using adjusted *p*-value < 0.05 and |log_2_FC| > 1.5 (see Materials and Methods). Comparison of RT-qPCR and RNA-Seq values on 10 genes presenting different patterns of expression in BAT93 and JaloEEP558 revealed a high correlation for these genes (Pearson *r* = 0.95), further confirming the reliability of RNA-Seq data (Fig. S2).

### Global impact of *X. phaseoli* pv. *phaseoli* on the transcriptome of common bean

**Fig. 2:**
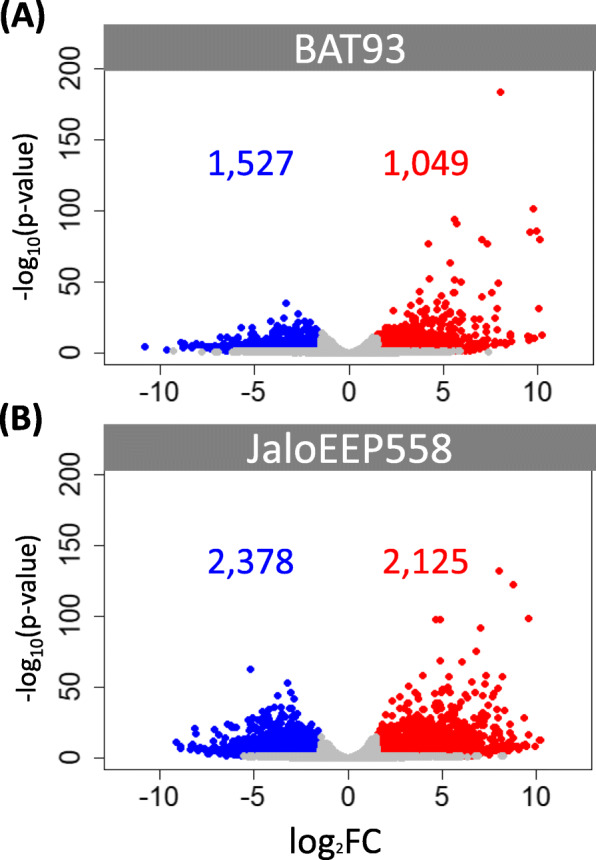
Global impact of strain CFBP6546R on the transcriptomes of BAT93 and JaloEEP558. Volcano plots represent the distribution of DEGs (adjusted *p*-value < 0.05, |log_2_FC| > 1.5) 2 days after inoculation of CFBP6546R on BAT93 (**a**) and JaloEEP558 (**b**). Each dot represents a gene either being upregulated (red), downregulated (blue) or non-differentially expressed (grey)

**Fig. 3:**
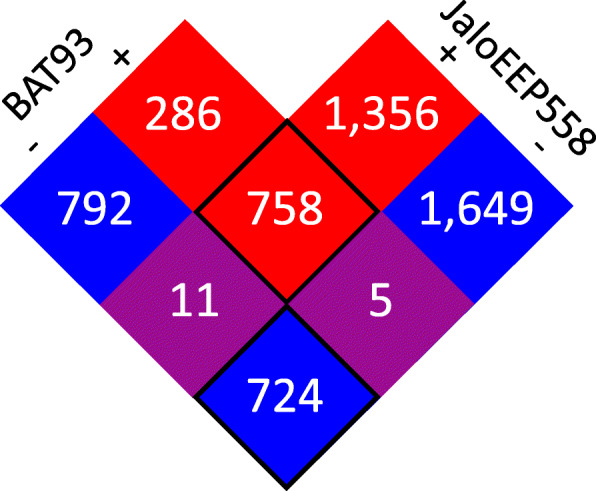
Venn diagram depicting the numbers of DEGs in BAT93 and JaloEEP558 during the interaction with strain CFBP6545R*.* Induced genes are highlighted in red while repressed genes are in blue. Purple squares represent genes with opposite responses in BAT93 and JaloEEP558. The core transcriptome corresponding to genes simultaneously induced or repressed in both genotypes is framed in black

**Fig. 4:**
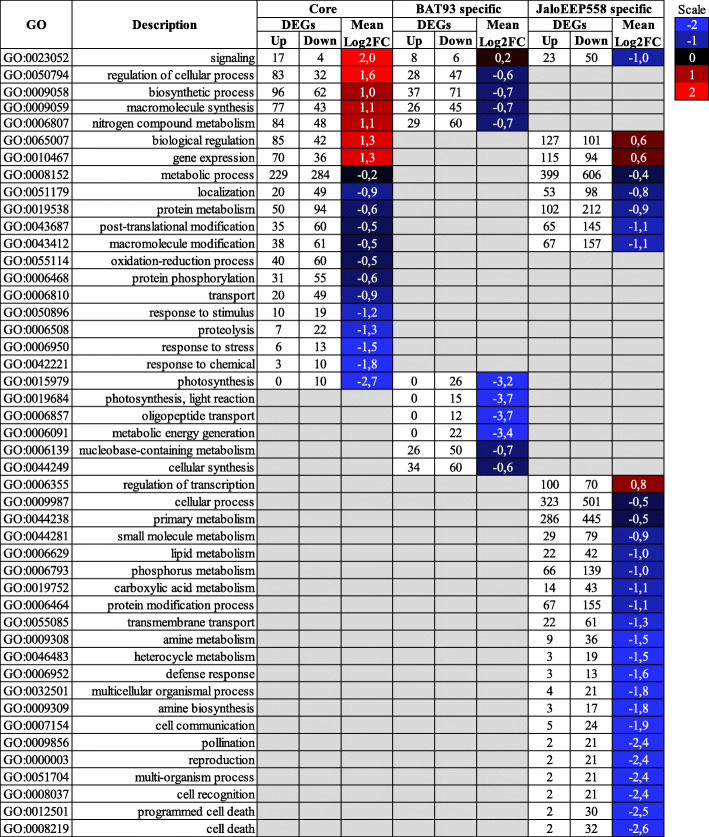
Enrichment tests on the core, the BAT93-specific and the JaloEEP558-specific transcriptomes. Enrichment tests were performed using the Parametric Analysis of Gene set Enrichment (PAGE) analysis on AgriGO v2 [59]. GO terms were considered enriched when comprising a minimum of 10 genes and presenting a False Discovery Rate (FDR) below 0.05. Enriched GO terms presented here were summarized using REVIGO [58]. Mean log_2_FC corresponds to the mean expression calculated using the log_2_FC from all DEGs within each GO. Grey boxes correspond to non-enriched GO terms

The susceptible genotype initiated a more intense and diverse biological response than the resistant genotype. Indeed, differential expression analysis identified 2576 and 4503 differentially-expressed genes (DEGs) in BAT93 and JaloEEP558 respectively, which represents 12 and 22% of the total homologs, respectively (Fig. 2). Three groups of DEGs could be defined (Fig. 3). First, the core transcriptome consisting of 1482 genes simultaneously induced (758) or repressed (724) in both genotypes. Then, two specific transcriptomes, consisting of 1094 genes specifically induced (291) or repressed (803) in BAT93, and of 3021 genes specifically induced (1367) or repressed (1654) in JaloEEP558. Enrichment tests identified 83, 32 and 126 gene ontology (GO) terms enriched in the core, the BAT93-specific and the JaloEEP558-specific transcriptomes, respectively (Table S4). To remove redundancy and poorly-informative GO terms, we focused our analysis on biological process GO terms summarized using REVIGO [58]. This analysis highlighted 20, 11 and 29 GO terms enriched in the core, the BAT93-specific and the JaloEEP558-specific transcriptomes, respectively (Fig. 4). We hypothesized that the core transcriptome was representative of the general response of common bean to *X. phaseoli* pv. *phaseoli*, while the differences observed between the two specific transcriptomes likely reflected the differences in resistance or susceptibility observed between both genotypes faced to *X. phaseoli* pv. *phaseoli*.

Resistance was linked to downregulation of photosynthesis while susceptibility was linked to downregulation of defenses. In BAT93, the most significantly enriched GO terms were related to photosynthesis (Table S4), with a large majority of repressed genes (Fig. 4), suggesting that the resistant genotype strongly suppresses production of primary energy. On the other hand, the most significantly enriched GO terms in JaloEEP558 were related to cell death (Table S4), with a majority of repressed genes annotated as *R* genes from the NLR family. Together with the fact that genes belonging to the GO term “defense responses” were predominantly repressed in this genotype (Fig. 4), this suggests that global defense responses are suppressed in the susceptible genotype. Interestingly, “signaling” was enriched in the specific transcriptomes of both BAT93 and JaloEEP558 as well as in the core transcriptome. DEGs belonging to “signaling” were mostly upregulated in the core transcriptome, while specific transcriptomes comprised both upregulated and downregulated genes. This suggests that common signaling pathways were induced in both genotypes, while other were specifically up- or downregulated in one genotype or another.

GO analysis done using up- and downregulated genes separately highlighted different GO terms than when using the whole dataset of DEGs (Table S5). It pointed out an upregulation of RNA metabolism and gene expression, as well as in nitrogen compound metabolic process in JaloEEP558, while the later was downregulated in BAT93.

### Detailed differences between resistant and susceptible genotypes

**Fig. 5:**
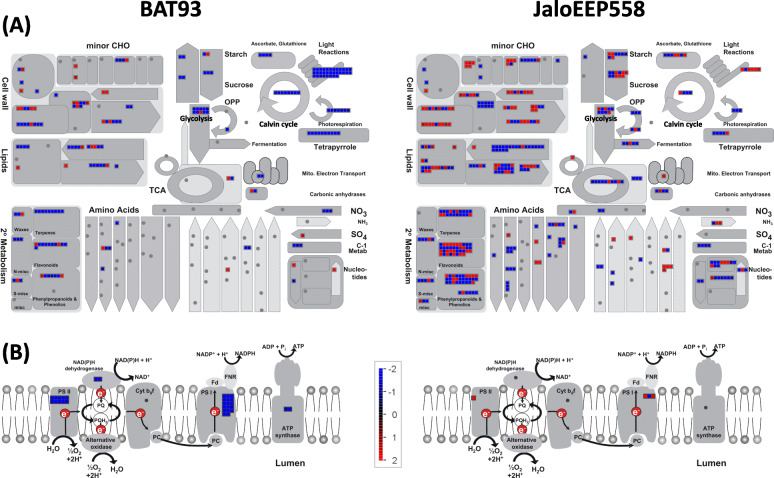
MapMan overview of DEGs in metabolic pathways (**a**) and the photosynthetic electron transport chain (**b**). DEGs from the specific transcriptomes of BAT93 (left) or JaloEEP558 (right) are represented by squares colored in blue (repressed) or red (induced) following the scale bar displaying changes in gene expression values in log_2_FC (in the center)

MapMan visualization gave a detailed overview of the differences between the resistant and susceptible genotypes (Fig. 5). In accordance with the GO analysis, Wilcoxon Rank Sum Test obtained with MapMan showed that genes related to photosynthesis were enriched in the BAT93-specific transcriptome, while defense genes related to biotic stress and signaling (RLK and PR genes) were enriched in the JaloEEP558-specific transcriptome (Table S6). Additionally, genes related to cell wall modification, ethylene signaling pathway and fatty acid metabolism were specifically enriched in JaloEEP558. To analyze the differences between both genotypes in more detail, we generated lists of genes from different classes (see Materials and Methods) and performed a KEGG analysis of hormonal signaling pathways (Table S7, Fig. S3).

#### NLR and RLK genes

We observed a specific repression of 30 NLR genes in the susceptible genotype while only one was specifically repressed in BAT93 (Table S7). Additionally, two NLR genes were specifically induced in JaloEEP558, while one (Phvul.006G056500) was both induced in BAT93 and repressed in JaloEEP558, thus appearing as a good candidate for being involved in CBB resistance. The RLK family presented a more complex pattern than NLRs, with a large number of RLK genes specifically induced or repressed in each genotype. More RLKs were repressed than induced in both genotypes, with 11 and 40 specifically induced and 34 and 125 specifically repressed in BAT93 and JaloEEP558, respectively (Table S7). As a result, more RLKs were differentially-expressed in the susceptible genotype than in the resistant genotype.

#### Kinases

A majority of kinases other than RLKs was induced in the core transcriptome (15/17, Table S7). In particular, four genes encoding calcium-dependent protein kinases (CDPKs) and calcineurin B-like protein-interacting protein kinases (CIPKs) were induced in both genotypes, indicating that genes linked to calcium signaling were induced in common bean during the interaction with *X. phaseoli* pv. *phaseoli* whatever the outcome (resistance or disease). In the JaloEEP558-specific transcriptome, a large modulation of the expression of kinases occurred, which followed the same trend of induction (20/29) than what was observed in the core transcriptome. On the other hand, BAT93-specific transcriptome was less impacted and a majority of kinases (10/12) was repressed.

#### Transcription factors

A larger induction of transcription factors (TFs) was specifically observed in JaloEEP558 (137/268) compared to BAT93 (28/91, Table S7). TFs from the APETALA2/ethylene response factor (AP2/ERF) superfamily presented the most differences between both genotypes (Fig. S3). Indeed, much more AP2/ERF genes were specifically induced in JaloEEP558 (32) than in BAT93 (4). In the meantime, less AP2/ERF genes were repressed in JaloEEP558 (6) than in BAT93 (8). This indicates a specific induction of AP2/ERF genes in the susceptible genotype 48 h post-inoculation. Similar patterns were observed for the MYB family, although with smaller differences between both genotypes compared to AP2/ERFs (Fig. S3). Other major TF families such as WRKY and GRAS were more impacted (i.e. both more induced and more repressed) in JaloEEP558 than in BAT93.

#### Lipid metabolism

Lipid metabolism was more impacted in the susceptible genotype (72 specific DEGs) than in the resistant genotype (23 specific DEGs, Table S7). Significantly, a large majority of genes involved in fatty acid synthesis (20/21) was specifically repressed in JaloEEP558 (Fig. 5a, Table S7). Some genes encoding proteins involved in lipid degradation, such as Alpha/beta hydrolases were also repressed in JaloEEP558, while others were upregulated, indicating that lipid degradation was impacted in different ways in the susceptible genotype [60]. On the other hand, fatty acid desaturases were mostly repressed in BAT93. Notably, the homolog of fatty acid desaturase 8 (*FAD8*, Phvul.006G068600) was both induced in JaloEEP558 and repressed in BAT93. These enzymes could play a role in defense activation [61, 62], hormone synthesis [63], increased membrane fluidity and influence membrane properties of chloroplasts [64]. Here, repression of these genes in the resistant genotype could be linked to the induction of plant defenses.

#### Photosynthesis and sugar metabolism

A global downregulation of photosynthesis and sugar metabolism occured in the resistant compared to the susceptible genotype (Fig. 5a). In particular, genes linked to the biosynthesis of photosystems I and II, the NAD(P) H deshydrogenase and the ATP synthase where largely repressed in BAT93 (Fig. 5b). By contrast, in JaloEEP558 three genes linked to the photosynthetic electron transport chain were induced. This result indicated a specific repression of the photosynthetic electron transport chain in the resistant genotype. More generally, the whole reactions taking place in the chloroplast appeared down-regulated in the resistant genotype, including the Calvin cycle, tetrapyrrole synthesis, photorespiration, the ascrobate and glutathione redox pathways (Fig. 5a). Sucrose and starch biosynthesis and degradation, and glygolysis were also specifically repressed in BAT93 (Fig. 5a).

#### Cell wall

Susceptibility was linked to the induction of cell wall modification and repression of cellulose biosynthesis while resistance was linked to the repression of genes involved in cell wall modification. Indeed, most genes involved in cell wall modification and degradation were induced in JaloEEP558 and repressed in BAT93. This was the case of xyloglucan endotransglycosylases, expansins, pectin methylesterase inhibitors, and glycosyl hydrolases (Fig. 5a and Table S7). On the other hand, genes involved in cellulose biosynthesis such as cellulose synthases were repressed in JaloEEP558.

#### Hormone signal transduction

Resistance and susceptibility were marked by opposite hormone signal transduction pathways. Indeed, resistance was linked to upregulation of SA signaling, while susceptibility was linked to upregulation of the ethylene pathway and downregulation of the SA and cytokinin pathways (Fig. S4). Several genes involved in resistance signaling were specifically repressed in JaloEEP558 such as the homolog of PATHOGENESIS-RELATED 1 (*PR1*, Phvul.006G197100) that was also specifically induced in BAT93 [65]. *PR1* being a marker of SA accumulation, these results suggest that upregulation of the SA pathway occurred in the resistant genotype while it was suppressed in a susceptible context. Consistently, cytokinin signaling was suppressed in JaloEEP558. This is highlighted by the strong induction of a homolog of genes encoding type A Arabidopsis response regulators (A-ARR), which are negative regulators of cytokinin signaling and SA-dependent basal immunity [66]. As described in the MapMan enrichment analysis, many genes involved in ethylene signaling were specifically induced in the susceptible genotype, including AP2/ERF genes (Table S6, Fig. S3). This trend is confirmed by the induction of homologs from ETHYLENE RESPONSE (ETR) and ERF1/2 genes (Fig. S4). Genes involved in other hormonal signaling pathways (auxin, brassinosteroids, gibberellins or abscisic acid) also showed contrasting expression profiles between resistant and sensitive genotypes, although the differences observed were not strong enough to be conclusive.

### Candidate genes for resistance to CBB

To search for genes putatively responsible for the resistance observed in BAT93, we have cross-checked different pieces of information that may suggest the involvement of certain genes in resistance to CBB. The different criteria we searched for were (*i*) that there was a large difference of log_2_FC values between the susceptible and resistant genotypes, (*ii*) that the gene was induced in one genotype and repressed in the other, and (*iii*) that the gene colocalized with a locus of resistance to CBB.

#### Genes with high log_2_FC difference

Interestingly, 201 DEGs had a large difference of expression (|Δlog_2_FC| > 5) between BAT93 and JaloEEP558 (Table S8). Among those genes, 148 (74%) were more expressed in JaloEEP558 than in BAT93. This proportion was higher than what was observed for the whole dataset (52%), and this difference was even higher when focusing on the top 50 genes with the largest |Δlog_2_FC|, among which 46 had a higher log_2_FC in JaloEEP558 than in BAT93 (Table S3). This indicates that the greatest differences observed between the two genotypes were due to genes strongly induced in JaloEEP558 and/or strongly repressed in BAT93. Of these 148 genes, the Heat Shock Protein (HSP) family was the most represented with 13 members, four of which belonged to the top 10 genes more expressed in JaloEEP558 than in BAT93. Significantly, three genes from the Lateral Organ Boundaries (LOB) family belonged to the list of 148 genes. Two of these *LOB* genes (Phvul.007G195100 and Phvul.008G257400) were ranked second and 15th of the most induced genes in JaloEEP558, suggesting that the induction of *LOB* genes is important for susceptibility. On the other hand, 53 genes had a higher log_2_FC in BAT93 than in JaloEEP558 (Table S8). A large proportion of these genes belonged to families related to resistance such as RLKs (7), NLRs (2) and PRs (2), indicating that a classical resistance response occurred in BAT93.

#### Genes with opposite reactions to *X. phaseoli* pv. phaseoli

**Fig. 6:**
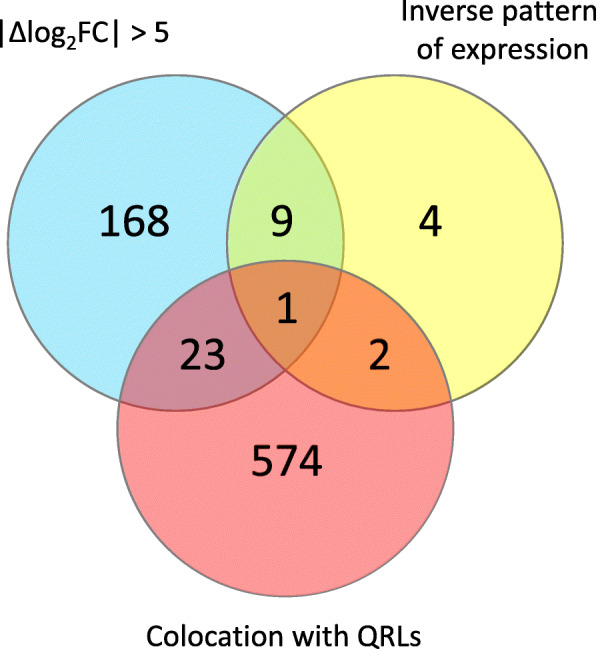
Venn diagram showing the numbers of DEGs potentially linked to resistance to CBB. The blue circle represents DEGs with a high Δlog_2_FC between BAT93 and JaloEEP558 (|Δlog_2_FC| > 5), the yellow circle represents DEGs with an inverse expression pattern between BAT93 and JaloEEP558 and the red circle represents DEGs that co-located with one of the four QRLs against CBB, described in the progeny of BAT93xJaloEEP558 [50]

Only 16 DEGs were simultaneously induced in one genotype and repressed in the other one (Table S9). Among those, 11 were both repressed in BAT93 and induced in JaloEEP558, suggesting that they contribute to either suppressing defenses and/or promoting disease (Fig. 3). Of those, six had also a |Δlog_2_FC| > 5, which encoded different proteins including a plasmodesmata-located protein (Phvul.001G229800), an HSP (Phvul.001G039700), a Lipid-Transfer Protein (Phvul.008G137100), a kinase (Phvul.008G081300), a pectin methylesterase inhibitor (Phvul.002G318500) and a sulfite exporter from the TauE/SafE family (Phvul.001G061000) (Table 2, Fig. 6). On the other hand, five genes were both induced in BAT93 and repressed in JaloEEP558, suggesting that they positively regulate resistance to CBB. Four of these genes had also a |Δlog_2_FC| > 5, including a placenta-specific 8 (PLAC8) family gene (Phvul.003G265800), an S-ribonuclease binding protein (SBP) family gene (Phvul.009G119200), a PR gene (Phvul.006G197100), and an ovate family gene (Phvul.009G057100).

**Table 2:** Candidate genes for resistance to CBB. Grey cases indicate non differentially-expressed genes (i.e. genes with −1.5 < log_2_FC < 1.5 and/or adjusted *p*-value ≥0.05)

Gene ID	Annotation	log_**2**_FC BAT93	log_**2**_FC JaloEEP558	|Δlog_**2**_FC| > 5	Inverse pattern of expression	Colocation with QRLs
Phvul.001G039700	17.6 kDa class II HSP	-1,67	8,75	Yes	Yes	–
Phvul.001G061000	Sulfite exporter TauE/SafE family	-1,86	3,23	Yes	Yes	–
Phvul.001G229800	plasmodesmata-located protein 7	-5,58	5,80	Yes	Yes	–
Phvul.002G212000	HSP20-like chaperones superfamily	-2,96	7,52	Yes	–	Yes
Phvul.002G231500	HSP20-like chaperones superfamily	-3,20	9,04	Yes	–	Yes
Phvul.002G243500	GDSL-like Lipase/Acylhydrolase superfamily	-8,35	-2,79	Yes	–	Yes
Phvul.002G249800	RmlC-like cupins superfamily	-3,81	1,43	Yes	–	Yes
Phvul.002G318500	Pectin methylesterase inhibitor	-1,86	3,43	Yes	Yes	–
Phvul.003G265800	PLAC8 family protein	3,25	-4,07	Yes	Yes	–
Phvul.005G014200	NLR	-1,02	-8,31	Yes	–	Yes
Phvul.005G034000	cytokinin oxidase/dehydrogenase 6	−9,67	−0,86	Yes	–	Yes
Phvul.005G044600	NAC (No Apical Meristem) domain transcriptional regulator superfamily	0,46	5,50	Yes	–	Yes
Phvul.005G049700	LOB 4	−1,25	4,23	Yes	–	Yes
Phvul.005G058600	MLP-like protein 43	4,19	−2,73	Yes	–	Yes
Phvul.005G085800	light-harvesting chlorophyll B-binding protein 3	−5,41	−0,30	Yes	–	Yes
Phvul.005G097600	plasmodesmata callose-binding protein 5	1,33	−3,99	Yes	–	Yes
Phvul.005G097800	bZIP transcription factor	−1,98	3,92	Yes	–	Yes
Phvul.006G197100	Pathogenesis-related 1 protein	4,85	−1,52	Yes	Yes	–
Phvul.007G013000	Eukaryotic aspartyl protease family	−5,87	1,45	Yes	–	Yes
Phvul.007G018600	FAD/NAD(P)-binding oxidoreductase family	−1,88	2,98	–	Yes	Yes
Phvul.007G024800	Major facilitator superfamily	1,05	−4,98	Yes	–	Yes
Phvul.007G030300	receptor serine/threonine kinase, putative	−1,32	−6,34	Yes	–	Yes
Phvul.007G038400	CYCLIN D3;1	−7,76	−2,01	Yes	–	Yes
Phvul.007G049700	cysteine-rich RLK 29	−1,88	1,68	–	Yes	Yes
Phvul.007G051300	cysteine-rich RLK 25	0,49	−5,54	Yes	–	Yes
Phvul.008G081300	Protein kinase superfamily	−3,96	3,96	Yes	Yes	–
Phvul.008G137100	Lipid-transfer protein	−3,68	5,63	Yes	Yes	–
Phvul.009G030900	Unknown protein	−5,53	0,56	Yes	–	Yes
Phvul.009G057100	ovate family protein 7	4,64	−4,99	Yes	Yes	Yes
Phvul.009G080200	17.6 kDa class II HSP	−3,10	4,47	Yes	–	Yes
Phvul.009G084400	Integrase-type DNA-binding superfamily	−0,53	7,55	Yes	–	Yes
Phvul.009G094000	plant natriuretic peptide A	−0,53	8,66	Yes	–	Yes
Phvul.009G097500	mitotic-like cyclin 3B	−5,85	−0,65	Yes	–	Yes
Phvul.009G106200	proline extensin-like receptor kinase 1	−2,56	2,82	Yes	–	Yes
Phvul.009G119200	SBP family	1,54	−4,90	Yes	Yes	–

#### Genes within QRLs

We positioned the four CBB QRLs previously identified in BAT93 on the bean reference genome and extracted the DEGs from specific transcriptomes located within these putative QRLs (Table S10). A total of 600 DEGs were retrieved in these four CBB QRLs, among which 24 (4.0%) had a |Δlog_2_FC| > 5, which is similar to the ratio observed in the whole transcriptomic data where 201 out of 5581 DEGs (3.6%) had a |Δlog_2_FC| > 5 (Fig. 6). In addition, 12 and 21% of the total genes were differently expressed in the QRLs of BAT93 and JaloEEP558, respectively, which is not different from what was observed for the whole transcriptome (Fig. 2). Thus, no specific pattern of expression could be observed in the QRLs compared to the rest of the common bean genome. Among the genes in QRLs with |Δlog_2_FC| > 5, the three most repressed genes in JaloEEP558 were one NLR gene (Phvul.005G014200) and two RLK genes (Phvul.007G051300 and Phvul.007G030300). Thus, these three genes represent good candidates for CBB resistance. The previously described HSP (Phvul.002G231500, Phvul.002G212000 and Phvul.009G080200) and LOB (Phvul.005G049700) genes specifically induced in JaloEEP558 or repressed in BAT93 were also retrieved in these QRLs.

Only one gene presented the three characteristics of being induced in BAT93 while repressed in JaloEEP558, having a |Δlog_2_FC| > 5 and locating in a QRL to CBB. This gene (Phvul.009G057100) is located within the QRL associated to marker *D0157* on chromosome 9. It belongs to the ovate family, which is involved in plant growth regulation and can suppress elongation [67–69].

## Discussion

In this work, we used RNA-Seq data to investigate the transcriptomic response of resistant and susceptible common bean genotypes during their interaction with *X. phaseoli* pv. *phaseoli.* Importantly, our work provided novel whole genome sequence data of two parental lines of a reference population (BAT93 x JaloEEP558) used in many genetic and genomic studies, including mapping of disease resistances [70–78].

A global trend was that the proportion of DEGs was higher in the susceptible genotype (22%) than in the resistant genotype (12%). This result highlights that susceptibility results in a larger reprogramming of gene expression than resistance, which was not surprising since a similar trend was observed in many other studies describing plant transcriptomic responses to diverse plant pathogens including fungi, bacteria and viruses [79–83].

In the core transcriptome, the induction of genes from the CIPK and CDPK families suggests that both genotypes are able to perceive *X. phaseoli* pv. *phaseoli* through Ca^2+^ signaling [84–87]. Following this hypothesis, the susceptibility to CBB observed in JaloEEP558 would result from inhibition of PTI by bacterial effectors rather than from non-detection by PRRs. In accordance with this, the susceptible genotype displayed a large repression of genes putatively involved in PTI such as RLKs, as well as defense response and NLR genes. This indicates that successful bean colonization by *X. phaseoli* pv. *phaseoli* is linked to suppression of plant defenses and reflects a potential involvement of bacterial effectors.

Pathogenic bacteria from the genus *Xanthomonas* are often described as hemibiotrophic [88]. Here, apparition of necrotic tissues suggested that *X. phaseoli* pv. *phaseoli* shifted from biotrophic to necrotrophic from 8 to 15 DPI. The defense response to biotrophic and hemibiotrophic pathogens is usually regulated by SA while defense responses to necrotrophic pathogens classically involve JA and ethylene [13, 89]. Here, a global induction of the SA pathway was linked to resistance, suggesting that an adapted SA response is effective in BAT93 48 h after infection by *X. phaseoli* pv. *phaseoli*. On the other hand, susceptibility was linked to the induction of the ethylene pathway and repression of the SA pathway, which are often described as being antagonistic to each other [89]. The induction of the ethylene pathway is reminiscent of the observation that ethylene is implicated in increased symptoms in other *Xanthomonas*-plant pathosystems [90–92]. In this view, it is tempting to speculate that ethylene is involved in successful colonization by *X. phaseoli* pv. *phaseoli*, while SA is involved in common bean resistance to CBB.

The specific suppression of photosynthesis, sugar metabolism and other chloroplast-associated genes observed in BAT93 reflects a rather classical defense response. Indeed, similar trends have been described using transcriptomics in different interactions between plants and bacteria [93–96] and integration of transcriptomic data from different pathosystems led to the hypothesis that suppression of photosynthesis is part of the plant adaptive immunity [97]. In addition, several studies have shown that incompatible interaction is linked to a decrease in photosynthetic activity [98–101] and that inhibition of photosynthesis often leads to the accumulation of reactive oxygen species [102]. Supporting this, it is interesting to note that *Xanthomonas citri* pv. *citri* is able to counter the decrease in photosynthesis by mimicking a plant natriuretic peptide, leading to suppression of resistance in citrus leaves [103].

The plant cell wall plays an important role in plant immunity, both as a physical barrier against pathogens and by releasing signaling compounds known as DAMPs when altered [104]. Successful pathogens are usually able to degrade the plant cell wall to access nutrients. In JaloEEP558, specific induction of genes involved in cell wall modification, such as expansins, xyloglucan endotransglycosylases and glycosyl hydrolases, suggests that remodeling of cell wall occurred in the susceptible genotype [105, 106]. In accordance with this, early CBB phenotype usually corresponds to water-soaking symptoms involving the softening and loosening of the cell wall. In contrast, cell wall modification genes were repressed in BAT93, which could suggest that cell wall rigidification occurred in the resistant genotype, thus preventing bacterial progression. Modification of the cell wall is tightly linked to cell size and shape and to morphogenesis of plant organs [107]. Interestingly, transcription factors from the LOB and ovate families were found both among the most differentially expressed genes between BAT93 and JaloEEP558, and among the DEGs located in QRLs to CBB.

LOB is a family of plant-specific transcription factors with key roles in the regulation of plant organ development [108, 109]. Induction of *CsLOB1* by *Xanthomonas* in *Citrus sinensis* triggers cell expansion and is required for symptom development [57]. Induction of *LOB* genes can induce genes involved in cell wall modification such as pectin methylesterase inhibitors [110]. Here, the strong induction of *LOB* genes in JaloEEP558 could contribute to the induction of downstream genes involved in cell wall modification and to the development of symptoms. The *LOB* gene Phvul.005G049700 is located on chromosome 5 and colocates with a QRL linked to marker *D1081*, explaining 15% of the phenotypic vartiation [50]. Therefore, Phvul.005G049700 could putatively act as a negative regulator of resistance to CBB.

On the other hand, the ovate family member Phvul.009G057100 was strongly induced in the resistant genotype and repressed in the susceptible genotype. This gene could positively contribute to CBB resistance as it colocates with a QRL on chromosome 9 that is linked to marker *D0157*, and explains 13% of the phenotypic variation [50]. Consistent with this hypothesis, ovate family members act as transcriptional repressors involved in the suppression of cell growth and elongation as well as in the regulation of secondary cell wall and vascular development [67–69]. However, to our knowledge, the ovate family has not so far been described as playing any role in plant-pathogen interaction.

Altogether, our analyses pointed out different molecular pathways appearing important for either promoting disease in the susceptible genotype or triggering immunity in the resistant genotype. The genes involved in these pathways were scattered throughout the whole common bean genome, which reflects the complexity of CBB resistance. In particular, large clusters of dozens of NLR genes exist at common bean subtelomeres [111–114] but the 30 NLRs repressed in JaloEEP558 following *X. phaseoli* pv. *phaseoli* infection did not correspond to the specific repression of one of these clusters. Thus, no particular locus was unveiled as being responsible for CBB resistance. To summarize, resistance was linked to suppression of photosynthesis and sugar metabolism and induction of the SA pathway, while susceptibility was linked to downregulation of plant defenses and upregulation of the ethylene pathway and AP2/ERF transcription factors as well as genes involved in cell wall modification.

*Xanthomonas* bacteria possess transcription activator-like effectors (TALE) that are type III effectors able to induce the transcription of genes by specifically binding to the promoter of plant susceptibility genes and recruiting the transcription machinery [115]. Nine different TALE-encoding genes and alleles have been described in CBB agents [116]. Strain CFBP6546R used in this study bears *tal19I* and *tal18H* [116]. Interestingly, most of the pathways induced in the susceptible genotype or repressed in the resistant genotype were previously described as being induced by TALEs to promote disease [117]. For example, the best-characterized TALE targets so far are *SWEET* genes that encode sugar exporters presumably providing nutrients for the pathogen [118–121]. *SWEET* gene induction by TALE has been described in the interaction of *Xanthomonas* with rice [121–127] cassava [128] and cotton [129]. According to our results, it is tempting to speculate that the suppression of photosynthesis and sugar metabolism observed in the resistant genotype could lead to the reduction of sugar production by the plant cells, thus contributing to resistance by depriving bacteria of sugar. Interestingly, one *SWEET* gene (Phvul.009G134300) was specifically repressed in the resistant genotype, while another one (Phvul.002G203600) was specifically induced in the susceptible genotype. However, Phvul.002G203600 was not predicted as a target of TAL19I or TAL18H [116]. Thus, but it seems that its induction was not due to the action of TALEs.

Other TALE targets include different types of TFs from the AP2/ERF [130], bHLH [54, 131] or LOB [57, 132, 133] families, which is reminiscent of what was observed here in the susceptible genotype. Interestingly, these targets are often linked to cell wall reorganization and modification of the plant cells shape. In pepper, the TALE AvrBS3 from *Xanthomonas campestris* pv. *vesicatoria* induces the bHLH TF UPA20, which leads to the hypertrophy of leaf cells [131]. In tomato, AvrHah1 from *X. gardneri* targets another bHLH TF whose induction upregulates the expression of a pectate lyase responsible for the apparition of water soaking symptoms [54]. In citrus, the induction of *CsLOB1* by different TALEs from *X. citri* pv. *citri* or *X. citri* pv. *aurantifolii* is required for the apparition of canker symptoms due to hyperplasia and rupture of the epidermis in infected tissue [57, 132, 133]. Here, the JaloEEP558-specific induction of *LOB* homologs and other genes involved in cell wall degradation and modification suggests that *X. phaseoli* pv. *phaseoli* employs mechanisms similar to what was observed in tomato and citrus. The parallel observed between our transcriptomic data and TALE targets in different plant species suggests that TALE evolution was driven by the necessity to target pre-existing susceptibility hubs in plants. This also raises the question as to whether *AP2*/*ERF*, *bHLH*, pectate lyases, or *LOB* homologs could constitute direct targets for TALEs from *X. phaseoli* pv. *phaseoli* in common bean. Thus, searching for *X. phaseoli* pv. *phaseoli* TALE targets in common bean would bring valuable knowledge on the molecular interactions between common bean and *X. phaseoli* pv. *phaseoli*.

## Conclusions

The analyses presented here bring novel information about the transcriptomic response of common bean to *X. phaseoli* pv. *phaseoli* attack, and may help to better understand the mechanisms underlying resistance to CBB in common bean. The global trends observed here lead to the hypothesis that the transcriptome of susceptible varieties is largely manipulated by the bacterium, while resistant varieties retain control of their transcriptome, increase signaling and adapt their metabolism for defense purposes.

## Methods

### Plant materials, bacterial strains and growing conditions

The original seeds from common bean cultivars BAT93 (Mesoamerican) and JaloEEP558 (Andean) were obtained from the Center for Tropical Agriculture (CIAT, Colombia) and are available under accession numbers G51294 and G9603, respectively (http://genebank.ciat.cgiar.org/genebank/main.do). Common bean cultivars were seeded in plastic pots (7 × 7 × 8 cm) containing pre-wetted soil. Plants were grown in a growth chamber at 23 °C/20 °C (day/night) with 80% relative humidity and a photoperiod of 16 h. Plants were watered every 2 days with water for the first 10 days, then with a nutrient solution of N-P-K (15–10-30). The day before inoculation, relative humidity and temperature were increased at 95% and 28 °C/25 °C (day/night) to provide adequate conditions for infection.

*X. phaseoli* pv. *phaseoli* strain CFBP6546R is a rifamycin-resistant derivative of strain CFBP6546. This strain was grown at 28 °C for 48 h on Trypticase Soy Agar (TSA) medium (17.0 g.L^− 1^ pancreatic digest of casein; 3.0 g.L^− 1^ enzymatic digest of soya bean; 5.0 g.L^− 1^ NaCl; 2.5 g.L^− 1^ K_2_HPO_4_; 2.5 g.L^− 1^ glucose; 15 g.L^− 1^ agar) supplemented by rifamycin (50 mg. L^− 1^), then at 28 °C for 24 h on TSA10 (a 1/10 dilution of TSA, except for agar maintained at 15 g.L^− 1^) supplemented by rifamycin (50 mg. L^− 1^) to obtain fresh bacterial cultures.

### Pathogenicity assays

Inoculations were performed at stage V1 (first trifoliolate leaf unfolded) by dipping the first trifoliate leaf for 30 s into bacterial suspensions calibrated at 1 × 10^7^ CFU.mL^− 1^ in sterile distilled water (CFU: colony-forming unit). Water-inoculated plants were used as control. For bacterial population sizes, at least three plants per condition were harvested at 2 h, 1, 5, 8, and 15 days post-inoculation (DPI). Each trifoliate leaf was ground individually for 2 min in a plastic bag supplemented with 10 mL of distilled water, using a Stomacher 80 (Seward, London, United Kingdom) at maximum power. Appropriate dilutions were plated on TSA10 supplemented by rifamycin (50 mg. L^− 1^) and incubated at 28 °C for 72 h before counting. At 8 and 15 DPI, symptom development was monitored by chlorophyll fluorescence imaging [134] at the PHENOTIC Seeds and Plants platform of the IRHS in Angers (France). Each leaflet was set in the dark for 30 min. Then, a first picture was taken under a modulated flash of light to measure basal fluorescence (F0) of the tissues, followed by another picture taken under a high flash of saturating light to measure the maximum fluorescence emission level (Fm). For each pixel, the maximum quantum yield of photosystem II photochemistry (Fv/Fm = (Fm-F0)/Fm) was calculated with Phenoplant (http://www.phenoplant.org) to discriminate diseased and healthy leaf areas [52]. Diseased areas were divided into different Fv/Fm clusters according to the intensity of the symptoms (impacted, wilted and necrotic) [52]. Pathogenicity assays were performed twice independently.

### Bean whole genome sequencing, assembly and annotation

Genomic DNA was extracted from young leaves and buds using the Nucleospin Plant II kit (Macherey-Nagel, Hoerdt, France). Paired libraries were prepared using the Illumina TruSeq Nano DNA Library prep kit, then sequenced at 2 × 150 bp on an Illumina HiSeq3000. Reads were trimmed to the first undefined base and assembled using SOAPdenovo software with k-mer size of 81 bp and max read length of 100 bp. Assembled contigs were gap filled and spurious assemblies were removed using redundancy search (97% identity, maximum overhang 50 bp). Scaffolds of minimum 1000 bp with at least 500 bp defined were retained. Annotation of the whole genome assembly was performed using EuGene-EP pipeline [135] using our BAT93 or JaloEEP558 RNA-Seq assemblies and four different protein databases as training sets for structural annotation: TAIR10, Swiss-Prot, UniProt plant subset and predicted proteins from the soybean (*Glycine max*) reference genome [136]. Gene completeness was assessed using BUSCO version 1.22 and the plantae dataset [137]. Gene functions presented here were determined by BLASTp (e-value ≤1 × 10^− 6^) on the *P. vulgaris* v2.1 reference genome from genotype G19833 [18]. Homologs between BAT93 and JaloEEP558 were searched for by reciprocal BLASTp (e-value ≤1 × 10^− 6^) of all predicted genes, and keeping only genes having the same best hit after both reciprocal BLASTp searches and the same best hit after BLASTp on the *P. vulgaris* reference genome. The four QRLs to CBB described in a BAT93 x JaloEEP558 progeny [50] were delimited by using the sequence of restriction fragment length polymorphism (RFLP) markers flanking these QRLs (Table S11), available at the PhaseolusGenes database (http://phaseolusgenes.bioinformatics.ucdavis.edu).

### RNA-Seq experiments

In order to obtain tissues with homogeneous and synchronous plant cells interacting with bacteria, leaflets from the first trifoliate leaves at stage V1 were detached from the plant, and vacuum-infiltrated for 2 × 1 min into a bacterial suspension of CFBP6546R strain at 1 × 10^8^ CFU.mL^− 1^ diluted in sterile distilled water, or pure sterile distilled water as control. Infiltrated leaflets were maintained in Petri dishes by dipping the petiole in water agar (0.7%) and incubated at 28 °C/25 °C (day/night) with a photoperiod of 16 h. Fourty-eigth hours after inoculation, 10 disks were sampled with a 1.3 cm diameter borer and immediately frozen in liquid nitrogen. Frozen disks were ground using a ball mill for 30 s at 25 Hz. Total RNA was extracted with the TRIzol® Plus RNA Purification Kit (Ambion, Applied Biosystems, Courtaboeuf, France) according to manufacturer’s recommendations, except that TRIzol® was used at 60 °C and that an additional DNase treatment was performed. The quantity and quality of total DNA-free RNA were evaluated using a NanoDropTM (Thermo Scientific, Wilmington, USA) and an ExperionTM chip (Bio-Rad, Hercules, CA, USA). Then, for each modality, RNA-Seq was performed on three independent biological replicates at the GeT-PlaGe core facility, INRAE Toulouse. RNA-Seq libraries have been prepared according to Illumina’s protocols using the Illumina TruSeq Stranded mRNA sample prep kit to analyze mRNA. Briefly, mRNAs were selected using poly-T beads. Then, RNAs were fragmented to generate double stranded cDNAs and adaptators were ligated to be sequenced. Eleven cycles of PCR were applied to amplify libraries. Library quality was assessed using a Fragment Analyser and libraries were quantified by qPCR using the Kapa Library Quantification Kit. All libraries were pooled and the whole pool was loaded into two sequencing lanes. RNA-Seq experiments have been performed on an Illumina HiSeq3000 using a paired-end read length of 2 × 150 pb with the Illumina HiSeq3000 sequencing kits. Total reads were mapped on the annotated genome sequences of BAT93 or JaloEEP558 and counted using the glint software (http://lipm-bioinfo.toulouse.inra.fr/download/glint). Only best-scores were taken into account, with a minimal hit length of 40 bp, a maximum of 5 mismatches and no gap allowed. Ambiguous matches with the same best score were removed. Principal component analysis and a Pearson correlation matrix were performed with R, using the number of reads per genes within each sample.

### Analysis of differentially-expressed genes

Differentially-expressed genes (DEGs) were determined using DeSeq2 with default normalization settings [138] and with adjusted *p*-value < 0.05 [139]. Three different log_2_FC thresholds were tested, at |log_2_FC| > 1, 1.5, or 2.0. DEGs were grouped according to three expression patterns. First, the core transcriptome consisting of genes simultaneously induced or repressed in both genotypes. Then, the BAT93-specific and JaloEEP558-specific transcriptomes consisting of genes specifically induced or repressed in BAT93 or JaloEEP558, respectively. Each gene set was used to perform an enrichment test based on Gene Ontologies (GOs) using the Parametric Analysis of Gene set Enrichment (PAGE) available on AgriGO v2.0 (http://systemsbiology.cau.edu.cn/agriGOv2) [59]. GO terms represented by a minimum of 10 genes and a False Discovery Rate (FDR) < 0.05 were considered significantly enriched. Enriched GO terms belonging to biological processes were summarized using REVIGO, a computational approach that summarizes long GO lists by reducing functional redundancies (http://revigo.irb.hr) [58]. Enriched GO terms from the three different log_2_FC thresholds were compared to each other, either by using all DEGs (Table S12). Consistency of enriched GO terms among the different log_2_FC tresholds was observed. Therefore, we used the intermediate log_2_FC threshold of 1.5 for all analyses. Functional annotations of DEGs were visualized using MapMan (http://mapman.gabipd.org) [140]. DEGs linked to hormone signaling were visualized on the pvu04075 pathway available on KEGG mapper [141] (https://www.genome.jp/kegg/mapper.html). Lists of DEGs associated with the cell wall or the metabolism of lipids were extracted using corresponding MapMan Bin codes. Transcription factors (TFs), kinase genes and RLKs used in this study were retrieved from the iTAK database (http://itak.feilab.net) [142]. The complete set of common bean NLRs was retrieved according to Richard et al. 2017a [143].

### RT-qPCR assays

To validate RNA-Seq results, RT-qPCR assays were performed on 10 selected genes representing different patterns of induction or repression in both bean genotypes. For each sample, cDNAs were synthetized from the total RNA using oligo (dT)15 primer with the M-MLV reverse transcriptase (Promega, Madison, WI, USA) according to manufacturer’s recommendations. RT-qPCR primers were designed using Primer3 [144] and checked for the absence of self or cross dimerization using Netprimer (https://www.premierbiosoft.com/netprimer, Table S13). Specificity and efficiency of each primer pair were checked by RT-qPCR and melting curves on a serial dilution of pooled cDNAs from the sample set. All primers produced a single peak with an efficiency ranging between 90 and 100%. RT-qPCR were performed with the MESA BLUE qPCR 2X MasterMix Plus for SYBR kit (Eurogentec, Seraing, Belgium) in a thermocycler with the following cycle: denaturation at 95 °C for 5 min, then 40 cycles of denaturation at 95 °C for 15 s followed by annealing and extending at 60 °C for 40 s. Relative expression levels were calculated using the 2^–ΔΔCt^ method for each gene and were normalized to the threshold cycle (Ct) values of the internal common bean reference genes *EF1*-*alpha* (Phvul.004G060000), *Actin 11* (Phvul.008G011000) and *Insulin-Degrading Enzyme* (*IDE,* Phvul.001G133200) [145]. For each modality, three technical replicates were made for each of the three biological replicates.

## Supplementary information

**Additional file 1: Figure S1** Statistical analysis of RNA-Seq data. Statistics were performed by using the total number of RNA-Seq reads mapped on the 20,787 homologs shared by BAT93 and JaloEEP558. Principal component analysis (**A**). Triangles and dots represent the barycenters of RNA-Seq data from BAT93 and JaloEEP558, respectively. Data from plants inoculated with H2O are in orange while data from plants inoculated with *Xanthomonas* strain CFBP6546R are in black. Pearson correlation matrix (**B**). Numbers represent the means of Pearson correlation coefficients calculated using three biological replicates per condition.

**Additional file 2: Figure S2.** Validation of RNA-Seq results by RT-qPCR analysis in BAT93 (**A**) and JaloEEP558 (**B**). Gene expression data were expressed according to the 2^–ΔΔCt^ method (Vandesompele et al. 2002) [145], relatively to three housekeeping genes: *Act11*, *EF1-α* and *IDE*, and to the value of water inoculated plants.

**Additional file 3 Figure S3** Numbers of DEGs in the different families of transcription factors. The list of common bean transcription factors was retrieved from the iTAK database (http://itak.feilab.net, Zheng et al. 2016) [142]. The numbers of induced and repressed genes are represented by red and blue bars respectively, in the specific transcriptomes of BAT93 (solid) or JaloEEP558 (hatched). Only families with at least 10 DEGs were represented.

**Additional file 4: Figure S4.** KEGG orthology map for plant hormone signal transduction. The KEGG orthology maps (pvu04075) of BAT93 (**A**) and JaloEEP558 (**B**) highlight DEGs that were induced (red), repressed (blue), or both induced and repressed (green) 48 h after inoculation with *Xanthomonas phaseoli* pv. *phaseoli*. The number of DEGs (colored numbers) and the average Log2FC (black numbers) are indicated above the boxes.

**Additional file 5: Table S1.** Number of genes shared among common bean genomes, as determined by reciprocal blastp (e-value <1E-6).

**Additional file 6: Table S2.** List of homologs shared by BAT93 and JaloEEP558.

**Additional file 7: Table S3.** List of DEGs in BAT93 and JaloEEP558 48 h after inoculation by *X. phaseoli* pv. phaseoli strain CFBP6546R.

**Additional file 8: Table S4.** Raw enrichment tests on the core, BAT93-specific or JaloEEP558-specific transcriptomes.

**Additional file 9: Table S5.** Enrichment tests on the core, BAT93-specific or JaloEEP558-specific transcriptomes, using induced or repressed DEGs separately.

**Additional file 10: Table S6.** List of enriched MapMan bins in the BAT93-specific and JaloEEP558-specific transcriptomes.

**Additional file 11.**

**Additional file 12: Table S8.** List of DEGs with |Δlog2FC| > 5.

**Additional file 13: Table S9.** List of DEGs with an inverse expression pattern between BAT93 and JaloEEP558.

**Additional file 14: Table S10.** List of DEGs in the four QRLs to CBB described in the progeny of BAT93xJaloEEP558 (Nodari et al., 1993) [50].

**Additional file 15: Table S11.** Genetic markers associated to the four QRLs to CBB described by Nodari et al. 1993 [the four QRLs to CBB described in the progeny of BAT93xJaloEEP558 (Nodari et al., 1993) [50].

**Additional file 16: Table S12.** Results of the enrichment tests according to the core, BAT93-specific or JaloEEP558-specific transcriptome and according to different log2FC threshold.

**Additional file 17: Table S13.** Primers used for RT-qPCR assays.

## Data Availability

Whole genome assemblies generated for this study have been submitted to GenBank under accession numbers JAAIFG000000000 (BAT93) and JAAIFH000000000 (JaloEEP58). Sequences and annotations are available at https://bbric-pipelines.toulouse.inra.fr/myGenomeBrowser?public=1. The raw RNA-Seq data and metadata have been submitted to the Gene Expression Omnibus under the accession number GSE155080 (SRA: SRP273448).

## Abbreviations

CBB: Common bacterial blight of bean
RNA-Seq: RNA sequencing
TALE: Transcription activator-like effector
PAMP/MAMP: Pathogen−/microbe-associated molecular pattern
PRR: Pattern-recognition receptor
RLK: Receptor-like kinase
PTI: PAMP-triggered immunity
ETI: Effector-triggered immunity
*R* gene/protein: Resistance gene/protein
NLR: Nucleotide-binding leucine-rich repeat
MAPK: Mitogen-activated protein kinase
SA: Salicylic acid
JA: Jasmonic acid
PR: Pathogenesis-related
PCR: Polymerase chain reaction
EU: European Union
QRL: Quantitative resistance locus
cDNA-AFLP: CDNA*-*amplified fragment length polymorphism
qRT-PCR: Quantitative real-time PCR
DPI: Days post inoculation
DEG: Differentially-expressed gene
GO: Gene ontology
CDPK: Calcium-dependent protein kinase
CIPK: Calcineurin B-like protein-interacting protein kinase
TF: Transcription factor
AP2/ERF: APETALA2/ethylene response factor
bHLH: Basic Helix-Loop-Helix
HSP: Heat-shock protein
MLO: Mildew resistance locus
EDS1: ENHANCED DISEASE SUSCEPTIBILITY 1
PR1: PATHOGENESIS-RELATED 1
JAZ1: JASMONATE ZIM-domain 1
FAD8: Fatty acid desaturase 8
LOB: Lateral Organ Boundaries
PLAC8: Placenta-specific 8
SBP: S-ribonuclease binding protein
DAMP: Damage-associated molecular pattern
FDR: False Discovery Rate
ETR: ETHYLENE RESPONSE
CIAT: Center for Tropical Agriculture
TSA: Trypticase Soy Agar
RFLP: Restriction fragment length polymorphism
PAGE: Parametric Analysis of Gene set Enrichment
CFU: Colony-forming units
gFM: Grams of fresh materials
