# Correction to: Common bean resistance to *Xanthomonas* is associated with upregulation of the salicylic acid pathway and downregulation of photosynthesis

**DOI:** 10.1186/s12864-020-07043-6

**Published:** 2020-09-23

**Authors:** Justine Foucher, Mylène Ruh, Anne Préveaux, Sébastien Carrère, Sandra Pelletier, Martial Briand, Rémy-Félix Serre, Marie-Agnès Jacques, Nicolas W. G. Chen

**Affiliations:** 1 IRHS, INRAE, AGROCAMPUS OUEST, Université d’Angers, SFR4207 QUASAV, 42, rue Georges Morel, F-49071 Beaucouzé, France; 2 CNRS, UMR 2594, Laboratoire des Interactions Plantes-Microorganismes (LIPM), F-31326 Castanet-Tolosan, France; 3 INRAE, US 1426, GeT-PlaGe, Genotoul, Castanet-Tolosan, France

**Correction to: BMC Genomics 21, 566 (2020)**

**https://doi.org/10.1186/s12864-020-06972-6**

Following the publication of the original article [1], it was reported that the correct image for Fig. 1 was missing. The correct Fig. 1 is provided here and has been added to the original article.

**Fig. 1:**
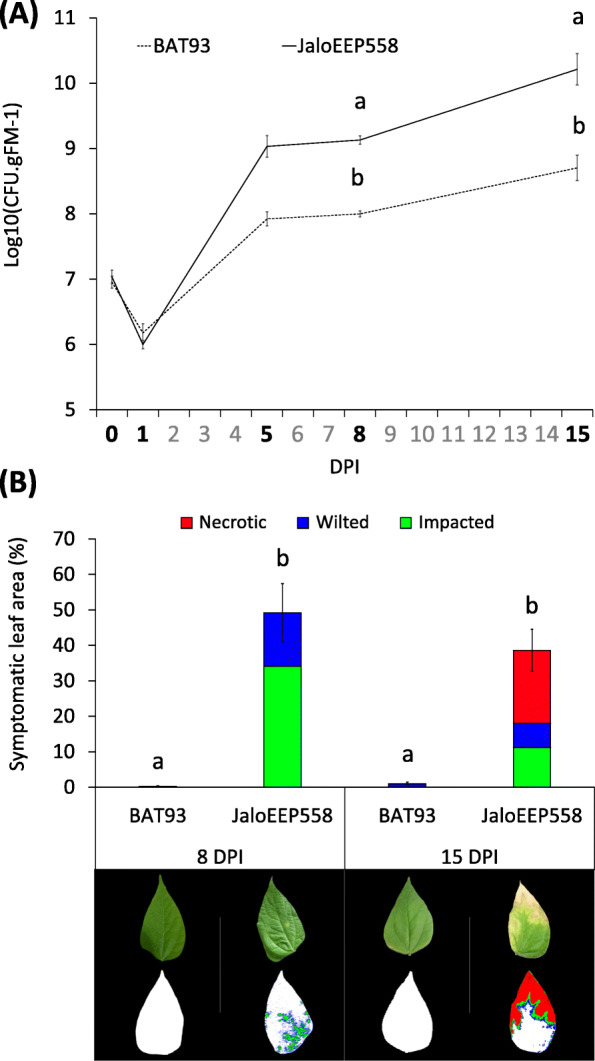
Pathogenicity of strain CFBP6546R on BAT93 and JaloEEP558. Bacterial population sizes over time on BAT93 (dotted line) and JaloEEP558 (full line) (a). Quantification of symptoms assessed by chlorophyll fluorescence imaging at 8 and 15 DPI (b). Total symptomatic areas corresponded to the sum of impacted, wilted and necrotic tissues defined by using Fv/Fm tresholds as previously compared to visual inspection [52]. Error bars represent the standard errors of the means for three biological replicates. Below the histogram are examples of leaflets presenting symptoms representative of each condition, obtained by chlorophyll fluorescence imaging (top) or visible imaging (bottom). Letters indicate significantly different groups (Mann-Whitney test, *p*-value < 0.05). CFU: colony-forming units. gFM: grams of fresh materials. DPI: days post inoculation

Furthermore, Table 2 was missing the indication of which values indicate non differentially-expressed genes (i.e. genes with − 1.5 < log2FC < 1.5 and/or adjusted *p*-value ≥0.05).

The original article has been updated.
