# Supplementary material for: Common bean resistance to Xanthomonas is associated with upregulation of the salicylic acid pathway and downregulation of photosynthesis
Source: BMC Genomics. 2020 Aug 18;21:566. doi: 10.1186/s12864-020-06972-6 (PMC7437933; doi:10.1186/s12864-020-06972-6)
Supplement: Supplementary file 1 — Additional file 1: Figure S1 Statistical analysis of RNA-Seq data. Statistics were performed by using the total number of RNA-Seq reads mapped on the 20,787 homologs shared by BAT93 and JaloEEP558. Principal component analysis (A). Triangles and dots represent the barycenters of RNA-Seq data from BAT93 and JaloEEP558, respectively. Data from plants inoculated with H2O are in orange while data from plants inoculated with Xanthomonas strain CFBP6546R are in black. Pearson correlation matrix (B). Numbers represent the means of Pearson correlation coefficients calculated using three biological replicates per condition. [file 12864_2020_6972_MOESM1_ESM.pptx]

## Slide 1
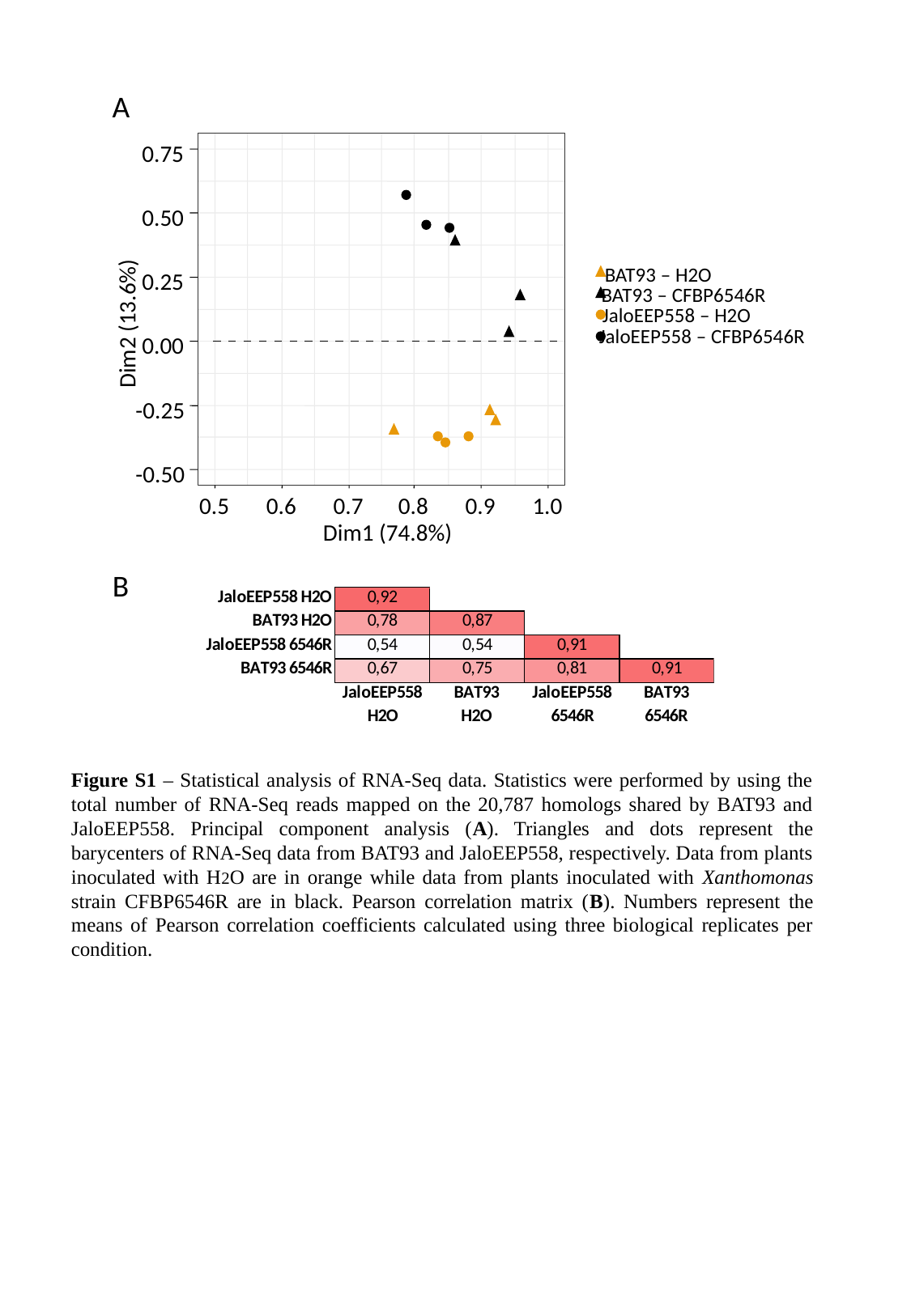

A
0.75
0.50
BAT93 – H2O
0.25
BAT93 – CFBP6546R
JaloEEP558 – H2O
Dim2 (13.6%)
JaloEEP558 – CFBP6546R
0.00
-0.25
-0.50
0.5
0.6
0.7
0.8
0.9
1.0
Dim1 (74.8%)
B
Figure S1 – Statistical analysis of RNA-Seq data. Statistics were performed by using the total number of RNA-Seq reads mapped on the 20,787 homologs shared by BAT93 and JaloEEP558. Principal component analysis (A). Triangles and dots represent the barycenters of RNA-Seq data from BAT93 and JaloEEP558, respectively. Data from plants inoculated with H2O are in orange while data from plants inoculated with Xanthomonas strain CFBP6546R are in black. Pearson correlation matrix (B). Numbers represent the means of Pearson correlation coefficients calculated using three biological replicates per condition.
