# Supplementary material for: Common bean resistance to Xanthomonas is associated with upregulation of the salicylic acid pathway and downregulation of photosynthesis
Source: BMC Genomics. 2020 Aug 18;21:566. doi: 10.1186/s12864-020-06972-6 (PMC7437933; doi:10.1186/s12864-020-06972-6)
Supplement: Supplementary file 2 — Additional file 2: Figure S2. Validation of RNA-Seq results by RT-qPCR analysis in BAT93 (A) and JaloEEP558 (B). Gene expression data were expressed according to the 2–ΔΔCt method (Vandesompele et al. 2002) [145], relatively to three housekeeping genes: Act11, EF1-α and IDE, and to the value of water inoculated plants. [file 12864_2020_6972_MOESM2_ESM.pptx]

## Slide 1
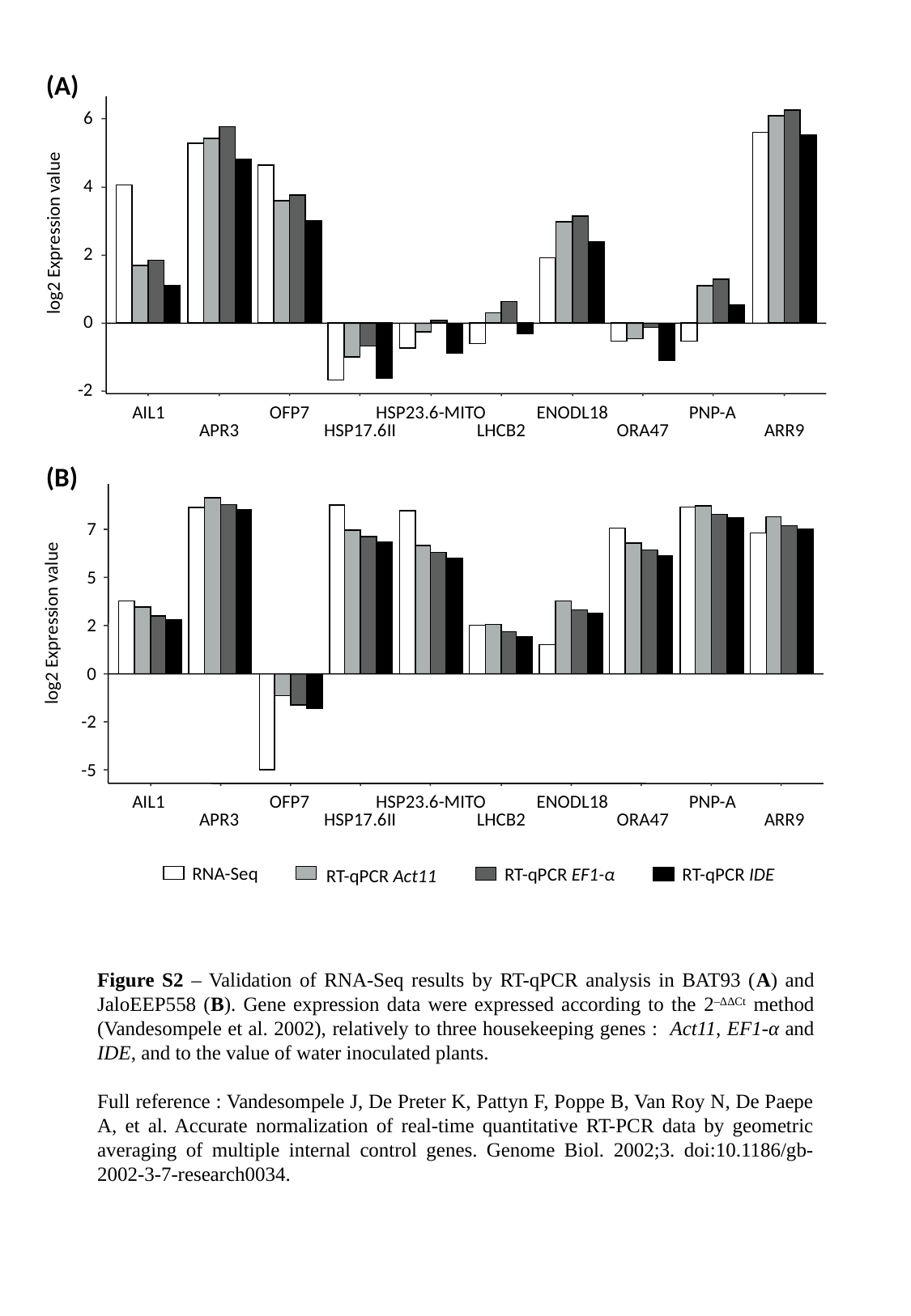

(A)
6
4
log2 Expression value
2
0
-2
AIL1
OFP7
HSP23.6-MITO
ENODL18
PNP-A
APR3
HSP17.6II
LHCB2
ORA47
ARR9
(B)
7
5
log2 Expression value
2
0
-2
-5
AIL1
OFP7
HSP23.6-MITO
ENODL18
PNP-A
APR3
HSP17.6II
LHCB2
ORA47
ARR9
RNA-Seq
RT-qPCR EF1-α
RT-qPCR IDE
RT-qPCR Act11
Figure S2 – Validation of RNA-Seq results by RT-qPCR analysis in BAT93 (A) and JaloEEP558 (B). Gene expression data were expressed according to the 2–ΔΔCt method (Vandesompele et al. 2002), relatively to three housekeeping genes : Act11, EF1-α and IDE, and to the value of water inoculated plants.
Full reference : Vandesompele J, De Preter K, Pattyn F, Poppe B, Van Roy N, De Paepe A, et al. Accurate normalization of real-time quantitative RT-PCR data by geometric averaging of multiple internal control genes. Genome Biol. 2002;3. doi:10.1186/gb-2002-3-7-research0034.
