# Supplementary material for: Common bean resistance to Xanthomonas is associated with upregulation of the salicylic acid pathway and downregulation of photosynthesis
Source: BMC Genomics. 2020 Aug 18;21:566. doi: 10.1186/s12864-020-06972-6 (PMC7437933; doi:10.1186/s12864-020-06972-6)
Supplement: Supplementary file 3 — Additional file 3 Figure S3 Numbers of DEGs in the different families of transcription factors. The list of common bean transcription factors was retrieved from the iTAK database (http://itak.feilab.net, Zheng et al. 2016) [142]. The numbers of induced and repressed genes are represented by red and blue bars respectively, in the specific transcriptomes of BAT93 (solid) or JaloEEP558 (hatched). Only families with at least 10 DEGs were represented. [file 12864_2020_6972_MOESM3_ESM.pptx]

## Slide 1
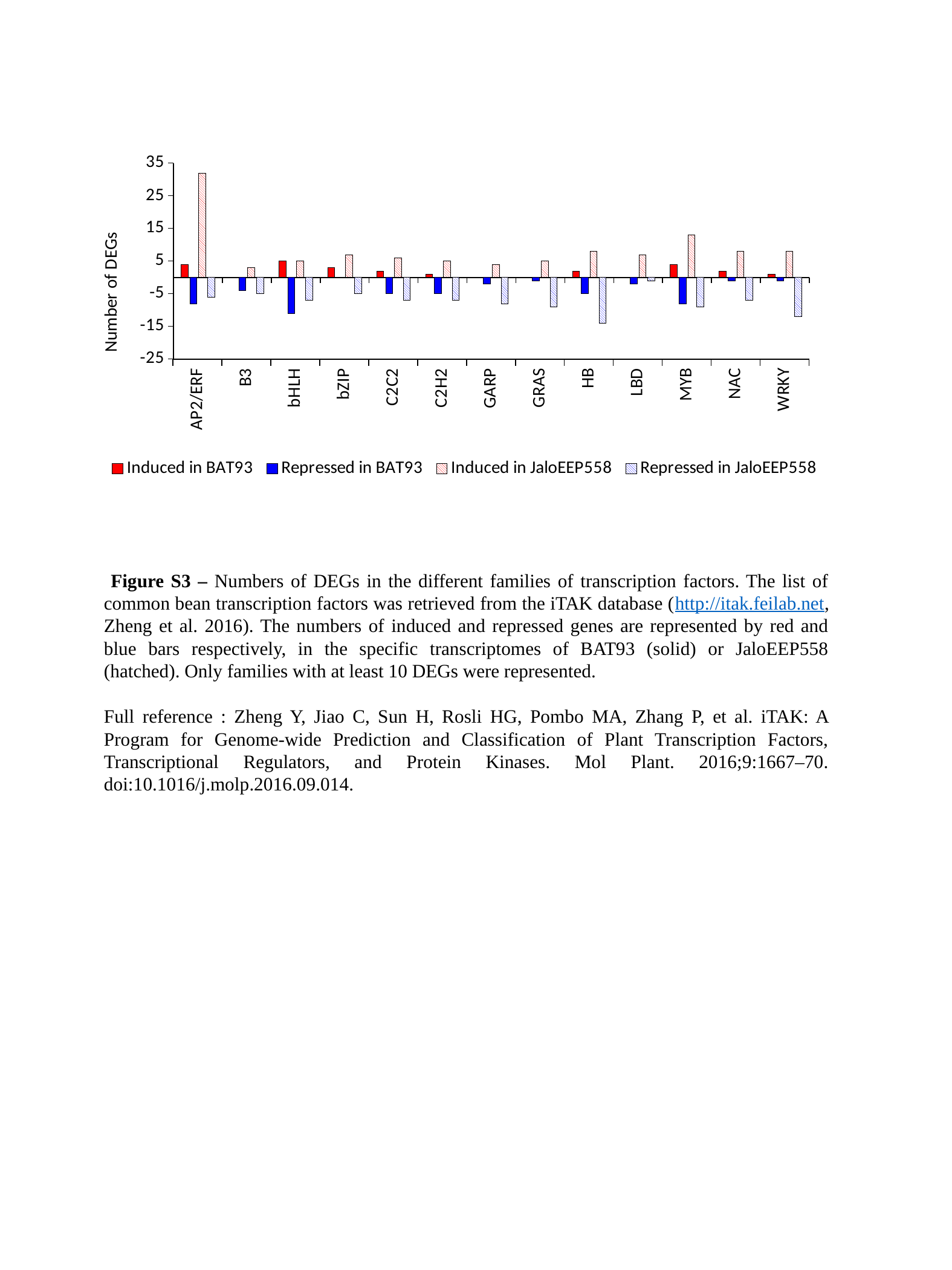

### Chart
| Category | Induced in BAT93 | Repressed in BAT93 | Induced in JaloEEP558 | Repressed in JaloEEP558 |
|---|---|---|---|---|
| AP2/ERF | 4.0 | -8.0 | 32.0 | -6.0 |
| B3 | 0.0 | -4.0 | 3.0 | -5.0 |
| bHLH | 5.0 | -11.0 | 5.0 | -7.0 |
| bZIP | 3.0 | 0.0 | 7.0 | -5.0 |
| C2C2 | 2.0 | -5.0 | 6.0 | -7.0 |
| C2H2 | 1.0 | -5.0 | 5.0 | -7.0 |
| GARP | 0.0 | -2.0 | 4.0 | -8.0 |
| GRAS | 0.0 | -1.0 | 5.0 | -9.0 |
| HB | 2.0 | -5.0 | 8.0 | -14.0 |
| LBD | 0.0 | -2.0 | 7.0 | -1.0 |
| MYB | 4.0 | -8.0 | 13.0 | -9.0 |
| NAC | 2.0 | -1.0 | 8.0 | -7.0 |
| WRKY | 1.0 | -1.0 | 8.0 | -12.0 |
 Figure S3 – Numbers of DEGs in the different families of transcription factors. The list of common bean transcription factors was retrieved from the iTAK database (http://itak.feilab.net, Zheng et al. 2016). The numbers of induced and repressed genes are represented by red and blue bars respectively, in the specific transcriptomes of BAT93 (solid) or JaloEEP558 (hatched). Only families with at least 10 DEGs were represented.
Full reference : Zheng Y, Jiao C, Sun H, Rosli HG, Pombo MA, Zhang P, et al. iTAK: A Program for Genome-wide Prediction and Classification of Plant Transcription Factors, Transcriptional Regulators, and Protein Kinases. Mol Plant. 2016;9:1667–70. doi:10.1016/j.molp.2016.09.014.
