# Supplementary material for: Common bean resistance to Xanthomonas is associated with upregulation of the salicylic acid pathway and downregulation of photosynthesis
Source: BMC Genomics. 2020 Aug 18;21:566. doi: 10.1186/s12864-020-06972-6 (PMC7437933; doi:10.1186/s12864-020-06972-6)
Supplement: Supplementary file 4 — Additional file 4: Figure S4. KEGG orthology map for plant hormone signal transduction. The KEGG orthology maps (pvu04075) of BAT93 (A) and JaloEEP558 (B) highlight DEGs that were induced (red), repressed (blue), or both induced and repressed (green) 48 h after inoculation with Xanthomonas phaseoli pv. phaseoli. The number of DEGs (colored numbers) and the average Log2FC (black numbers) are indicated above the boxes. [file 12864_2020_6972_MOESM4_ESM.pptx]

## Slide 1
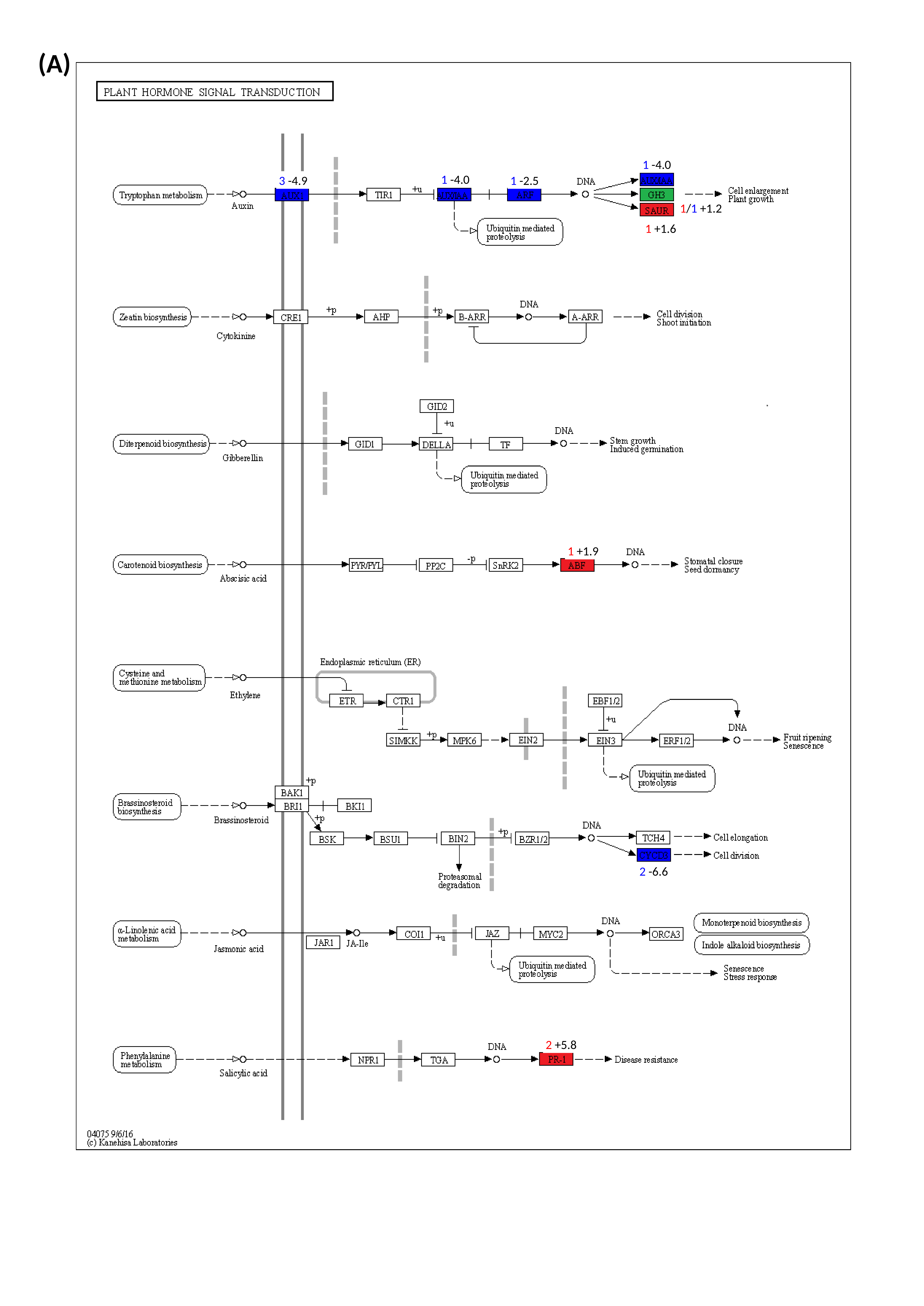

(A)
1 -4.0
1 -4.0
1 -2.5
3 -4.9
1/1 +1.2
1 +1.6
1 +1.9
2 -6.6
2 +5.8

## Slide 2
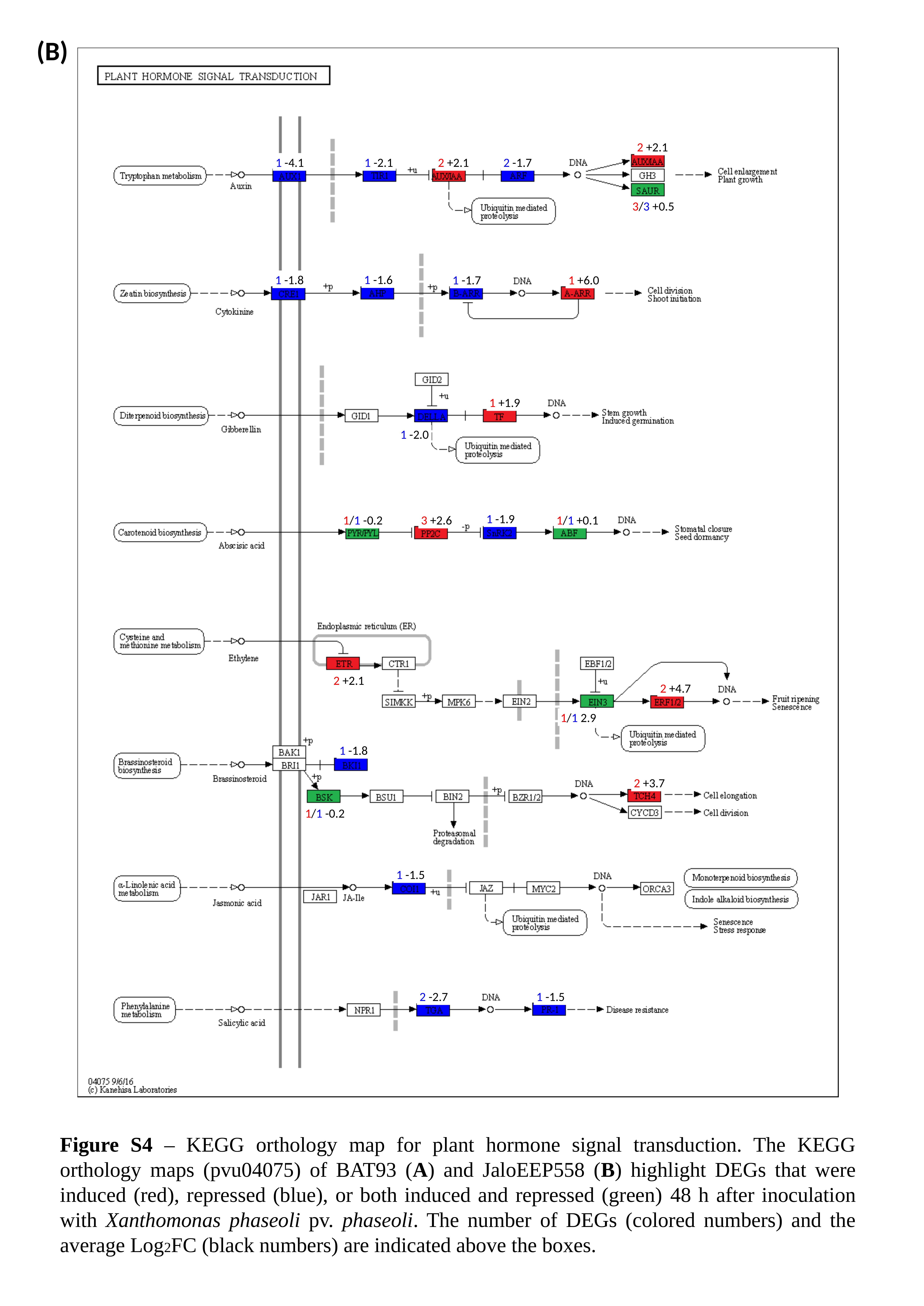

(B)
2 +2.1
2 +2.1
2 -1.7
1 -2.1
1 -4.1
3/3 +0.5
1 -1.6
1 -1.8
1 +6.0
1 -1.7
1 +1.9
1 -2.0
1 -1.9
1/1 +0.1
3 +2.6
1/1 -0.2
2 +2.1
2 +4.7
1/1 2.9
1 -1.8
2 +3.7
1/1 -0.2
1 -1.5
2 -2.7
1 -1.5
Figure S4 – KEGG orthology map for plant hormone signal transduction. The KEGG orthology maps (pvu04075) of BAT93 (A) and JaloEEP558 (B) highlight DEGs that were induced (red), repressed (blue), or both induced and repressed (green) 48 h after inoculation with Xanthomonas phaseoli pv. phaseoli. The number of DEGs (colored numbers) and the average Log2FC (black numbers) are indicated above the boxes.
